# Richmond Academy of Medicine and Surgery—Regular Meeting Held August 22, 1899

**Published:** 1899-10

**Authors:** 


### Richmond Academy of Medicine and Surgery—Regular Meeting Held August 22, 1899.

Dr. E. C. Levy, president, in the chair; Dr. Mark W. Peyser, reporter.

Dr. Virginius Harrison read a paper as folows:

### SHALL WE OPERATE IN EVERY CASE OF APPENDICITIS?

We find in nearly every journal something on the subject of appendicitis. This is because there is no subject of more importance to us, as doctors, and it is the so-called mild cases of appendicitis about which our profession differs more often in regard to the treatment. This will continue until our methods of diagnosis are perfected in regard to the severity of the conditions existing within the abdomen, enabling us to lay down exact rules for the varying cases.

Those who favor the medical or expectant treatment use various arguments for substantiating their belief, the chief of which are, the patient may recover if not operated on; if the patient gets worse rather than better within a day or two, then operate. Some advise us to wait until the attack has been recovered from, and operate in the interim. Taken as a rule, these statements may have had and did deserve some consideration a few years ago, when we had not the experience of today, which has so clearly demonstrated the advantage attained by an early operation.

Many have been the cases treated expectantly, and apparently with success, for the pain had subsided, the temperature and pulse had become either normal or nearly so, the rigidity of the abdominal muscles had relaxed, when suddenly symptoms of serious import arose, indicating suppurative peritonitis, or the rupture of a local abscess. Some cases are so mild, apparently, that after a few days' treatment we may doubt our diagnosis and render another, and not until a post-mortem has revealed the true condition of affairs do we appreciate how insidious the symptoms of appendicitis can be. A case of this kind has, I am told, occurred in surgical Richmond within the past two months.

Again, I have seen cases operated upon in which there was some doubt as to the existence of any serious lesion of the appendix, yet the operation taught us we had not operated too early. I recall a case in point; a young man had recurrent attacks so frequently that I advised the removal of the appendix. These attacks never kept him in bed more than one day. He never had a temperature over 100° F., nor was his pulse more than 80. There was always some little rigidity, though very slight. Before the last attack he skated until eleven o'clock p. m., and was taken the next morning at four o'clock with severe pain, which was promptly relieved by the action of a dose of Epsom salts. From these symptoms this surely could be classed as a mild case. I operated at one o'clock, and was much surprised to find a localized collection of pus ready to rupture and be followed by suppurative peritonitis.

I have seen all the conditions I have spoken of and many more illustrated many times over on the operating table. Nearly every operation for appendicitis impresses the fact upon me that early operation gives the patient the best chance of recovery. I honestly believe we will lose nothing by operating on every case as soon as we are sure of our diagnosis, provided there are not other diseased conditions that must engage our consideration. To use the words of Dr. Deaver: "Until the good Lord makes the belly wall transparent, it will be impossible to do other than guess as to the progress the appendiceal inflammation is making."

A few surgeons, and among them some high authorities, adhere to "the middle of the road" theory, advising that some patients be operated upon and others not; yet there are many bright luminaries in the surgical world, such as Murphy, Deaver, Treeves and others, who freely admit that they cannot tell what course any case of appendicitis will take, and advise the removal of the appendix; while the mortality should not be over two per cent. I envy the man who has the courage to act upon his honest convictions, provided these convictions have been made after a studious investigation of the subject.

When does the time-limit of a mild case end, or, in other words, how long should we wait before operating in case of mild symptoms? The time ends, in my opinion, when we have diagnosed the case as appendicitis. The damage is often done early in the attack. The two cases already referred to teach this. I could give you the history of a good many cases to demonstrate this fact and the correctness of my opinion; but, instead, shall quote the statistics of Dr. Will. J. Means, of Columbus, as given at the June meeting of the American Medical Association: * * * "In nine cases operated on in the first twenty-four hours after the attack, the appendix was ruptured in five and coprolites were escaping through the openings. In the four other cases the appendix was inflamed, the mucosa showed pathologic changes, and the surrounding tissues were more or less involved. In seventeen cases operated on within forty-eight hours, the appendix was gangrenous in ten. In twenty-three cases operated on in seventy-two hours, twenty of these were gangrenous in some portion. In twenty-five cases operated on after the third day, there were abscesses in twenty. What do we learn from these statistics? Surely, it is the lesson that the earlier we operate the less grave will be the conditions with which we will have to deal, and therefore the lowest rate of mortality will be had in the early operations." If the statistics given by Dr. Means were unique we might stop and meditate as to their value, but when we find that his experience coincides with the experience of nearly all of those who operate daily, and who have the opportunity of verifying their statements over and over again, we must accept their statements as true, and act accordingly, until some light hitherto withheld has illuminated the abdomen and directed us correctly as to which cases will go on to complete resolution and which will hasten on from bad to worse with a fatal termination.

While we are still in the dark as to the ultimate outcome of any case, does it not seem more reasonable and better to open a few bellies in one hundred cases that do not require it, and have all, or practically all, of the cases to get well, than to wait until the appendix has become perforated, or gangrenous, and involving all the surrounding tissues? This latter condition will entail a far more serious operation and be attended with a corresponding higher rate of mortality.

Doubtless patients have had attacks of appendicitis, recovered and never had a return of the attack. We all know that this is the exception, and not the rule. If these cases were labeled so that we could recognize them, there would be no diversity of opinion as to the treatment. As a rule, one attack is followed by another sooner or later. We do not know that the attack supposed to have been the primary attack was in reality the primary attack at all; the patient may have had several and have been treated for some other abdominal trouble. The symptoms are no criterion in determining the amount of damage done within the abdominal walls. I have seen a flat abdomen, a normal temperature and pulse accompanying pus in the abdomen.

I wish to speak briefly of two other classes of appendicitis, viz., the acute fulminating and those cases seen after the advent of suppurative peritonitis. No one will, I suppose, take issue with me when I say all cases of the acute fulminating variety should be operated upon immediately. Appendicitis has become such a common disease that this class is usually diagnosed by the family by the time we are called. They have learned the necessity of an operation in the majority of instances. The old doctrine "too late for the early operation and too early for the late operation" has no place here. As soon as the patient can be placed in suitable surroundings to be operated upon the more chance will there be of success. Even slight delay is fraught with great danger. An hour may be sufficient for this appendiceal inflammation to do irremedial damage and place the patient beyond the pale of successful surgery.

This brings me to the consideration of a class of cases that deserves a more careful consideration as to the needs of an operation. I refer to those seen after suppurative peritonitis has set in. What shall we do with them? If we do not open the abdomen, irrigate and drain the peritoneal cavity, we know that the patient is inevitably doomed. If we operate, the large majority of the patients will die, and surgery will suffer as a consequence. These are the cases that the surgeon who is working for good statistics will avoid if possible.

Is this right? Some of these cases have been saved. This being true, should it not be our duty to lend them our aid? For they are entitled to even the small chance of saving their lives if they desire it. The statistics of the surgeon, or the effect upon future surgery, amounts to nothing to them in comparison to their own lives. Again, by operating on these cases, we shall learn more of them, and may find a better method whereby we may be able to save a great many more than can be done by the present method.

In conclusion, I shall summarize by advising in all mild cases, when there are no complications of great moment, the use of "an ounce of prevention rather than a pound of cure," by operating just as soon as the diagnosis is complete. In the fulminating variety we all agree, I have no doubt, as to the advantage to be gained by the early operation. In those cases seen late, and in which suppurative peritonitis is present, without collapse, I think the patient is entitled to the very small chance given by the operation, after he has been fully advised as to the gravity of the situation.

[The discussion on this paper will be published in next issue. —Ed.]

